# Spectroscopic Evaluation of Glioma Grading at 3T: The Combined Role of Short and Long TE

**DOI:** 10.1100/2012/546171

**Published:** 2012-07-31

**Authors:** E. Kousi, I. Tsougos, E. Tsolaki, K. N. Fountas, K. Theodorou, I. Fezoulidis, E. Kapsalaki, C. Kappas

**Affiliations:** ^1^Medical Physics Department, University of Thessaly, Biopolis, 41110 Larissa, Greece; ^2^Neurosurgery Department, University of Thessaly, Biopolis, 41110 Larissa, Greece; ^3^Diagnostic Radiology Department, University of Thessaly, Biopolis, 41110 Larissa, Greece

## Abstract

*Purpose*. To evaluate the diagnostic value of 3T ^1^H-MRS in grading cerebral gliomas using short and long echo times. *Methods*. 1H-MRS was performed on 71 patients with untreated cerebral gliomas. Metabolite ratios of NAA/Cr, Cho/Cr, Cho/NAA, and mI/Cr were calculated for short and long TE and compared between low and high grade gliomas. Lipids were qualitatively evaluated. ROC analysis was performed to obtain the cut-off values for the metabolic ratios presenting statistical difference between the two glioma grades. *Results*. Intratumoral Cho/Cr at both TEs and long TE Cho/NAA were significantly different between low and high grade gliomas. Peritumoral NAA/Cr of both TEs, as well as long TE Cho/Cr and Cho/NAA ratios, significantly differentiated the two tumor grades. Diagnostic sensitivity of peritumoral short TE NAA/Cr proved to be superior over the other metabolic ratios, whereas intratumoral short TE Cho/Cr reached the highest levels of specificity and accuracy. Overall, short TE 1H-MRS reached higher total sensitivity in predicting glioma grade, over long TE. *Conclusion*. An advantage was found in using short TE over long TE 1H-MRS in the discrimination of low versus high grade gliomas. Moreover, the results suggested that the peritumoral area of gliomas may be more valuable in predicting glioma grade than using only the intratumoral area.

## 1. Introduction

 Gliomas are the most common primary neoplasms of the central nervous system (CSN), varying histologically from relatively benign primary brain tumors (e.g., astrocytomas) to more malignant grades (anaplastic astrocytomas, glioblastomas) with a survival probability beyond 5 years to be lower than 5% [1]. Prospective grading of primary cerebral gliomas is a hazardous endeavor but with a significant benefit in planning therapeutic approaches, as well as assessing the prognosis and response to therapy [2]. Histopathological assessment—the current reference standard for tumor grading—is accompanied by appreciable risks of morbidity and mortality [3] and inherent sampling errors may produce confusing results even for trained pathologists [2]. 

 Conventional magnetic resonance imaging (MRI) cannot always elucidate glioma heterogeneity and extent and sometimes can provide confusing or misleading features [4]. Proton magnetic resonance spectroscopy (^1^H-MRS) has been performed to improve the characterization of tumors and the surrounding normal appearing white matter (NAWM). It provides metabolic information regarding the tissue under study, complementing the anatomic information obtained with conventional MRI [5]. Typical ^1^H-MRS findings of cerebral gliomas include reduction of N-acetyl-aspartate (NAA), variable levels of creatine (Cr) and an elevation of choline (Cho), lactate (Lac) and lipids in proportion to glioma grade. Nevertheless, it is not always possible to distinguish among glioma grades (specifically in cases of grades II and III) regarding the metabolic findings of their intratumoral area [6, 7].

Therefore, the interest in terms of tumor differentiation has been redirected in the peritumoral area of such lesions [8–10]. The hypothesis is that the surrounding tissue of high grade gliomas is expected to be intensely infiltrated by tumoral cells when compared to low grade gliomas. 

Hence, ^1^H-MRS can potentially resolve ambiguities remaining after conventional MRI concerning tumor grade and extent and could reduce unnecessary biopsy procedures [11–16], reaching up to 91.1% specificity on predicting tumor type [17]. 

For a meaningful ^1^H-MRS procedure, spectroscopic data collection should be optimized and thus different parameters concerning signal acquisition may be varied. Those parameters determine not only the appearance of the spectrum but also the reliability of the metabolic information obtained. The time, at which an echo signal is being acquired (TE), is among the most important of such parameters and easy to modify. At present, TE used during in vivo ^1^H-MRS ranges between 18–288 msec. Short TE (18–45 msec) ^1^H-MRS allows the observation of several metabolites such as macromolecules (MMs), myo-inositol (mI) and mobile lipids [18]. However, short TE spectra show a large number of overlapping peaks and a strong MM/lipid-originated baseline, and data acquisition is prone to artifacts [18]. On the other hand, long TE (120–288 msec) spectra are less informative because resonances with short T2 may be lost [19]. Nevertheless, long TE signals are easier to analyze and lipid resonances (1.3 and 0.9 ppm) and MMs will not be the dominating components, facilitating the study of lactate (doublet at 1.33 ppm) and alanine (doublet at 1.47 ppm) as inverted peaks [20, 21]. Few investigators have assessed the contribution of both short and long TE ^1^H-MRS for glioma grading, collecting metabolic data only from the intratumoral area of such lesions [14, 22, 23]. To the best of our knowledge, no previous studies have evaluated both short and long TE metabolic findings of the peritumoral area for glioma grading.

For that reason, the purpose of the current study was to overall assess the contribution of short and long TE ^1^H-MRS at 3T in distinguishing high from low grade gliomas and particularly evaluate the hypothesis that there should be significant differentiation between the two tumor groups regarding the metabolic findings of their intratumoral as well as their peritumoral area. 

## 2. Methods and Materials

### 2.1. Patients

 Seventy-one patients (age range: 16–77) with untreated gliomas were studied before surgical biopsy or resection at our institution after given written informed consents. Histopathological diagnosis was obtained via either biopsy or resection and all tumors enrolled were graded according to the current World Health Organization (WHO) criteria. The biopsies were performed at locations similar to the voxel placement in ^1^H-MR spectroscopy. Nineteen patients were classified as low grade gliomas (8 oligodendrogliomas and 11 astrocytomas) and 52 as high grade gliomas (8 anaplastic astrocytomas, 3 anaplastic oligoastrocytomas, and 41 glioblastomas). 

### 2.2. MRI and ^1^H-MRS Protocols

MRI and ^1^H-MRS were performed on a 3-Tesla whole body unit (GE, Healthcare, Signa HDx) equipped with a birdcage head coil. 

### 2.3. Conventional MRI Protocol

Conventional MRI protocol included precontrast sagittal and transverse T1-weighted fast spin echo (FSE) (TR/TE = 700 msec/9.3 mssec), transverse T2-weighted FSE (TR/TE = 2,640 msec/102 msec), coronal T2-weighted FSE (TR/TE = 2,920 msec/102 msec), and transverse T2-weighted fluid attenuation inversion recovery (FLAIR) (TR/TE = 8,500 msec/130 msec). Diffusion-weighted MR imaging was performed via a single shot, spin echo, echo planar sequence with *b* value of 0, 1,000 ssec/mm^2^. Postcontrast isotropic 3D-spoiled gradient echo (3D-SPGR-TR/TE = 6.9 msec/2.1 msec, 12° flip angle, 240∗240 mm^2^ FOV, 136 slices with 1 mm thickness) and T1-weighted FSE (TR/TE = 700 msec/9.3 msec) axial images were also obtained.

### 2.4. ^1^H-MRS Protocol

^1^H-MRS data acquisitions using PROton Brain Exam (PROBE) Single Voxel (SV) spectroscopy and two dimensional-MRSI (2D-MRSI) were performed before contrast administration to avoid signal disturbance. Data were acquired using Point-RESolved Spectroscopy (PRESS) pulse sequence with automatic shimming and Gaussian water suppression. Measurement parameters used in SV scans were 1500/35 msec (TR/TE) and 128 signal acquisitions (Nacq), and voxel size was chosen to be not less than 3.375 cm^3^ for adequate SNR. Measurement parameters used in 2D-MRSI were 1,000/144 msec (TR/TE), 16 × 16 phase encoding steps, 10 mm section thickness and the field of view (FOV) size was adjusted to each patient's brain anatomy.

**Figure 1:**
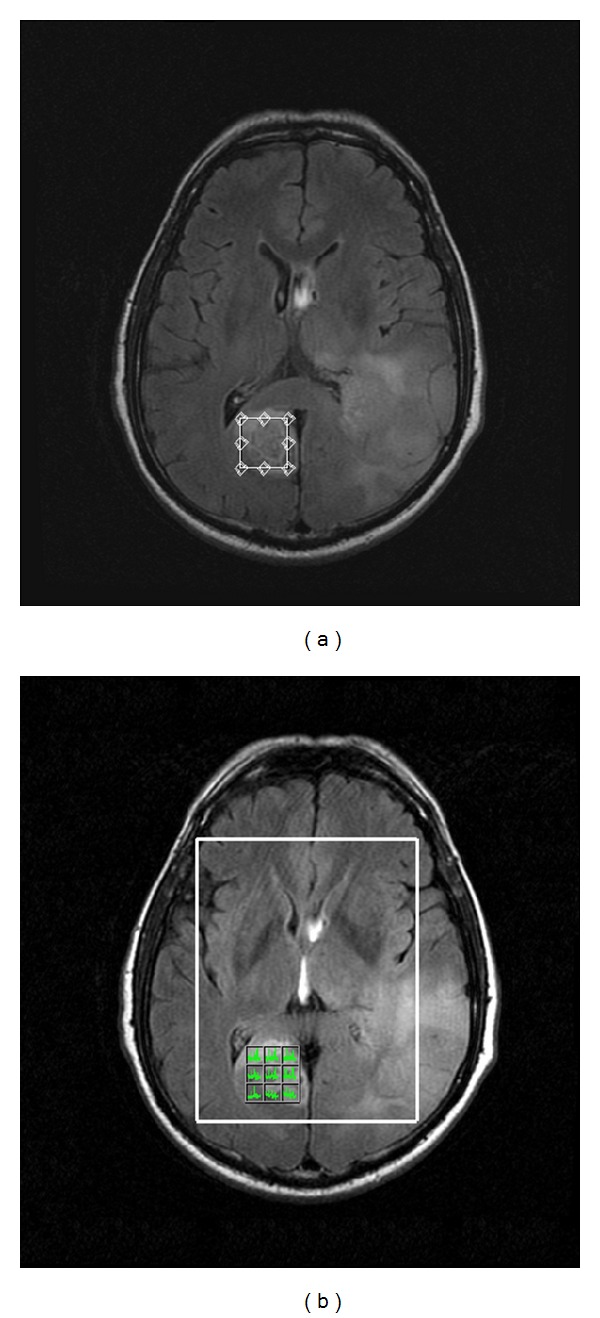
Intratumoral voxel prescription during SV (a) and 2D-MRSI (b) scan techniques, in a 77-year-old woman with a glioblastoma. For the two scan techniques, the voxel of interest has been placed in similar areas.

For both spectroscopic techniques, a rectangular ROI was localized by using the transverse T2-weighted FLAIR or T2-weighted FSE, sagittal T1-weighted FSE and coronal T2-weighted FSE imaging sequences. Spectra for each patient were acquired from the intratumoral, peritumoral, and contralateral regions of interest. The contralateral normal area (cNA) was used as the control spectrum. Within the tumor, the size and location of the voxel were carefully adjusted to include as much of the solid tumor portion as possible, avoiding the inclusion of obvious necrosis, cyst, hemorrhage, edema, calcification, and normal-appearing brain. For each patient, 3 SV scans were performed, one for each region of interest (intratumoral, peritumoral, cNA). Consequently, a 2D-MRSI scan was performed, and the spectra were collected from similar regions as those previously obtained from the SV scans (Figure 1) in order to have a direct metabolite comparison between the two ^1^H-MRS techniques. 

Spectral processing was based on a computer workstation by using the MR user interface (General Electric Healthcare). Postprocessing of the raw spectral data included baseline correction, frequency inversion, and phase shift.

### 2.5. Spectra Evaluation and Statistical Analysis

 The main metabolites identified by ^1^H-MRS are N-acetyl-aspartate (NAA) at 2.02 ppm, creatine (Cr) at 3.0 ppm, choline containing compounds (Cho) at 3.2 ppm and myo-inositol (mI) at 3.6 ppm. Concerning lipids and lactate (Lac) we qualitatively defined and estimated their sum (LL) between 0.9 and 1.3 ppm. 

For all the regions of interest (intratumoral, peritumoral, cNA), the metabolic ratios of NAA/Cr, Cho/Cr, mI/Cr, and Cho/NAA were calculated (mean ± SD).

 Data analysis was performed using the SPSS (v13) statistical software package. Nonparametric Mann-Whitney *U* tests were used to evaluate the significance in the metabolic ratio differences between high and low grade gliomas and between the affected (intratumoral, peritumoral) and the contralateral (cNA) areas. In order to identify the optimal cut-off values of the most discriminative metabolic ratios, receiver operating characteristic curve (ROC) analysis based on logistic regression models was performed. The efficacies of the parameters were assessed in terms of sensitivity and specificity. *P* values less than 0.05 were considered statistically significant.

## 3. Results

On short TE, the intratumoral metabolic ratio values for NAA/Cr, Cho/Cr, mI/Cr, and Cho/NAA were 1.04 ± 0.39, 1.52 ± 0.46, 0.85 ± 0.24, and 1.65 ± 1.08 for low grade gliomas and 1.22 ± 0.39, 1.90 ± 0.63, 0.90 ± 0.35, and 1.40 ± 0.60 for high grade gliomas. 

On long TE, the intratumoral metabolic ratio values of NAA/Cr, Cho/Cr, mI/Cr, and Cho/NAA were 1.29 ± 0.53, 2.17 ± 0.67,0.34 ± 0.14, and 2.02 ± 1.20 for low grade gliomas and 1.19 ± 0.44, 2.99 ± 1.18, 0.40 ± 0.27, and 2.93 ± 1.72 for high grade gliomas.

Concerning the peritumoral regions, the metabolic ratios of NAA/Cr, Cho/Cr, mI/Cr, and Cho/NAA on short TE were 1.65 ± 0.45, 0.83 ± 0.15, 0.61 ± 0.16, and 0.51 ± 0.09 for low grade gliomas and 1.30 ± 0.29, 1.00 ± 0.27, 0.55 ± 0.11, and 0.79 ± 0.31 for high grade gliomas. 

On long TE, peritumoral NAA/Cr, Cho/Cr, mI/Cr and Cho/NAA ratios were 1.74 ± 0.57, 1.26 ± 0.27, 0.24 ± 0.09, and 0.79 ± 0.32 for low grade gliomas and 1.34 ± 0.39, 1.58 ± 0.52, 0.21 ± 0.14, and 1.22 ± 0.63 for high grade gliomas.

 Additionally, metabolic ratios were calculated from the contralateral normal area (cNA), which is important to ensure metabolic ratios abnormality from the intratumoral and the peritumoral regions. 

The mean ± S.D. of the metabolic ratios concerning the intratumoral and the peritumoral areas and the cNA of low and high grade gliomas are summarized in Tables 1 and 2.

### 3.1. Intratumoral Region

Low grade gliomas demonstrated variable amounts of Cho elevation and NAA decrement. Specifically on both TEs applied, Cho was the dominant peak on 9/19 (47%) low grade glioma spectra, while the remainders exhibited only a mild Cho elevation. NAA was markedly reduced on 10/19 (53%) low grade gliomas, while the rest showed a moderate NAA decrease. 

 High grade gliomas exhibited a prominent Cho peak on 20/52 (38%) cases, while NAA was found markedly reduced on all high grade glioma spectra when short TE was applied. On the contrary, on long TE spectra, all high grade gliomas exhibited a prominent Cho peak, while NAA was greatly reduced or absent. 

mI was found to be increased for both tumor groups. 

**Figure 2:**
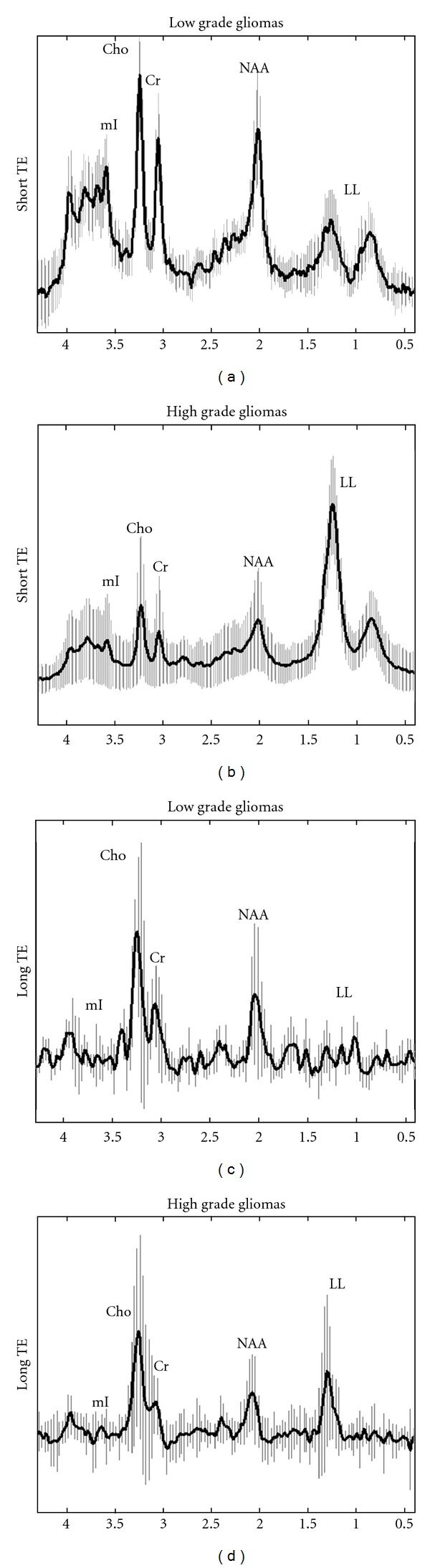
In vivo short TE ((a), (b)) and long TE ((c), (d)) mean spectra with their corresponding standard deviations (vertical lines) from the intratumoral area of low grade ((a), (c)) and high grade gliomas ((b), (d)).

Concerning the intratumoral area, the mean spectra of high and low grade gliomas for both TEs are illustrated in Figure 2.

**Figure 3:**
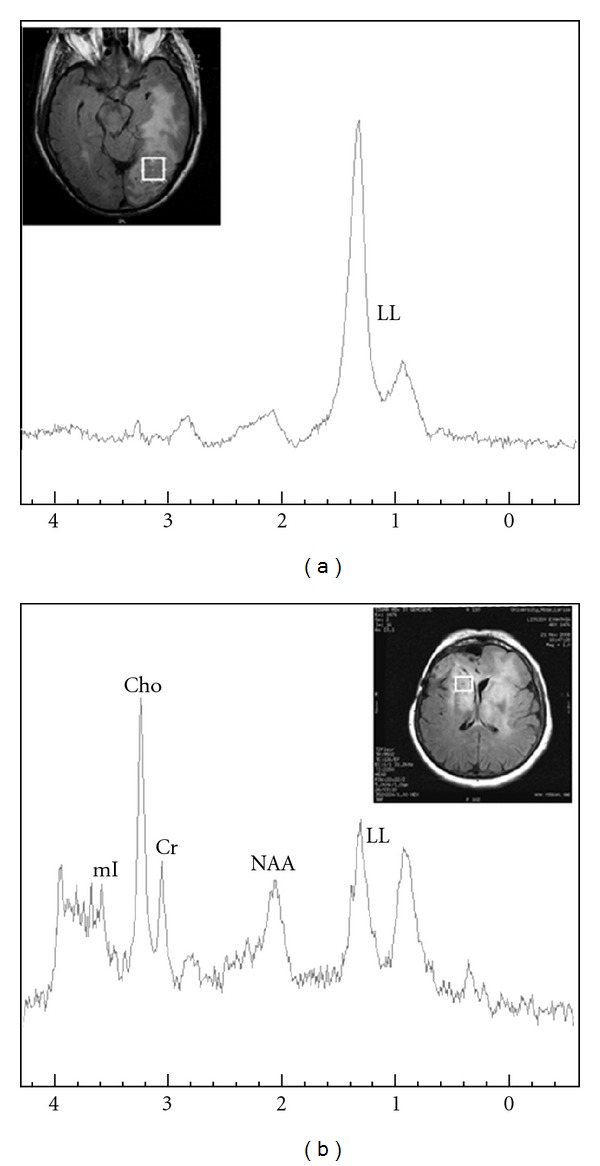
Short TE ^1^H-MR spectra of a 66-years-old male (a) and a 51-year-old female (b) with glioblastoma multiform and anaplastic astrocytoma, respectively. Spectra illustrate high grade glioma intratumoral patterns of (a) dominant LL peak and (b) smaller LL peak with clear evidence of all the other metabolites.

By qualitatively evaluating the LL peaks, when short TE was performed, high grade gliomas showed two spectroscopic patterns: (a) 30/52 (58%) cases exhibited a dominant LL peak in their spectra, without clear evidence of other metabolic peaks, and (b) 22/52 (42%) cases exhibited a variable amount of LL peaks. Typical spectra of both patterns are illustrated in Figure 3. 

**Figure 4:**
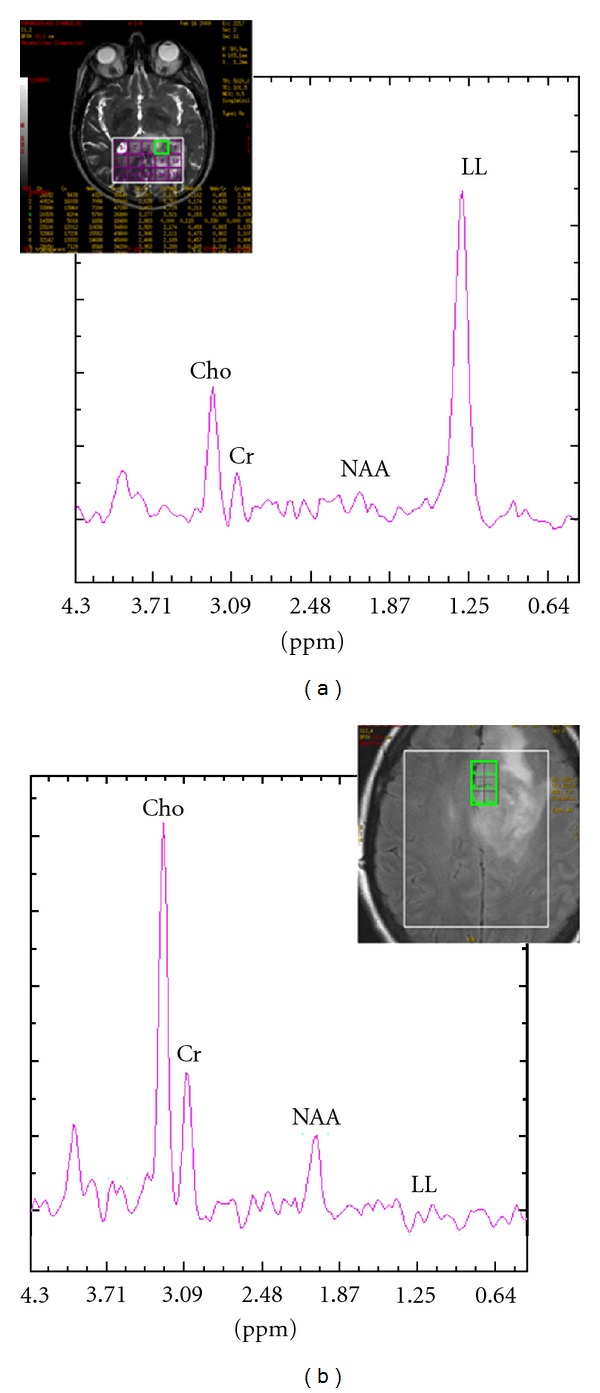
Long TE ^1^H-MRSI spectra of a 54-year-old male (a) and a 47-year-old female (b) with glioblastoma multiform and anaplastic oligoastrocytoma, respectively. Spectra illustrate high grade glioma intratumoral patterns of (a) high LL peak and (b) absence of LL peak respectively.

Similarly, when long TE was applied, two spectral patterns were also observed for high grade gliomas: (a) 20/52 (38%) demonstrated high LL peak while the remainders did not (b) (Figure 4). 

 LL peaks in low grade glioma spectra were within normal range for both TEs performed. 

All computed intratumoral metabolic ratios of low grade and high grade gliomas were statistically different from those of the cNA, revealing a distinct differentiation of the two tumor groups from the normal brain parenchyma. 

Comparing the intratumoral metabolic ratios, short TE Cho/Cr and long TE Cho/Cr and Cho/NAA ratios were significantly higher for high grade gliomas (Table 1). mI was observed to be increased for both glioma grades as mentioned above and hence that ratio did not significantly differentiate the two tumor groups. 

### 3.2. Peritumoral Region

In low grade gliomas, perilesional infiltration was observed in only 2 (10%) patients where Cho/Cr ratio was found to be elevated on the periphery of the lesion, either using short or long TE. Additionally, a mild NAA decrease was observed in the periphery of 17/19 (89%) low grade gliomas on both TEs. 

**Figure 5:**
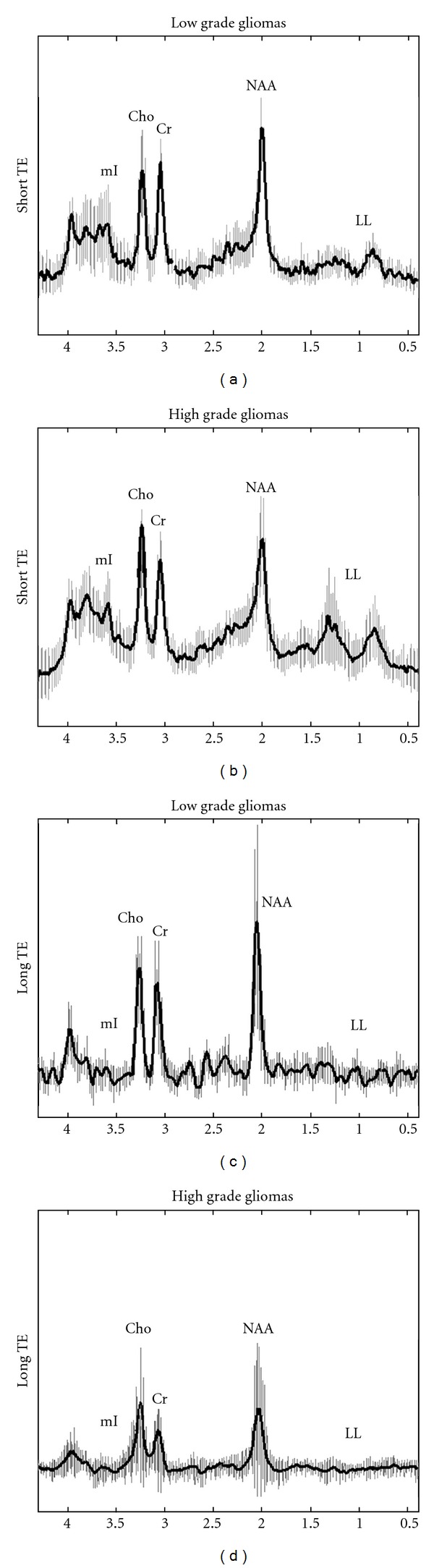
In vivo short TE ((a), (b)) and long TE ((c), (d)) mean spectra with their corresponding standard deviations (vertical lines) from the peritumoral area of low grade ((a), (c)) and high grade gliomas ((b), (d)).

High grade gliomas exhibited higher peritumoral Cho and lower NAA levels. Nevertheless, 4/52 (8%) high grade gliomas did not reveal evidence of tumor infiltration as Cho/Cr and NAA/Cr ratios from their periphery were within normal ranges for both TEs used. Mean spectra of the peritumoral area of low and high grade gliomas are illustrated in Figure 5.

Peritumoral NAA/Cr of either TEs, as well as long TE Cho/Cr, mI/Cr and Cho/NAA ratios of high grade gliomas, was statistically different from the corresponding control values of the cNA. 

On the contrary, only long TE mI/Cr from the peritumoral region of low grade gliomas was statistically different from the cNA, while the rest metabolic ratios were very close to the corresponding control values giving no statistically significant differences.

Hence, comparing the metabolic ratios in the peritumoral region of interest between the two glioma grades, NAA/Cr of both TEs was found to be significantly lower for high grade gliomas. Similarly, long TE Cho/Cr and Cho/NAA ratios were significantly higher for high grade gliomas (Table 1), reflecting the difference of the two tumor groups in invading adjacent brain parenchyma.

**Figure 6:**
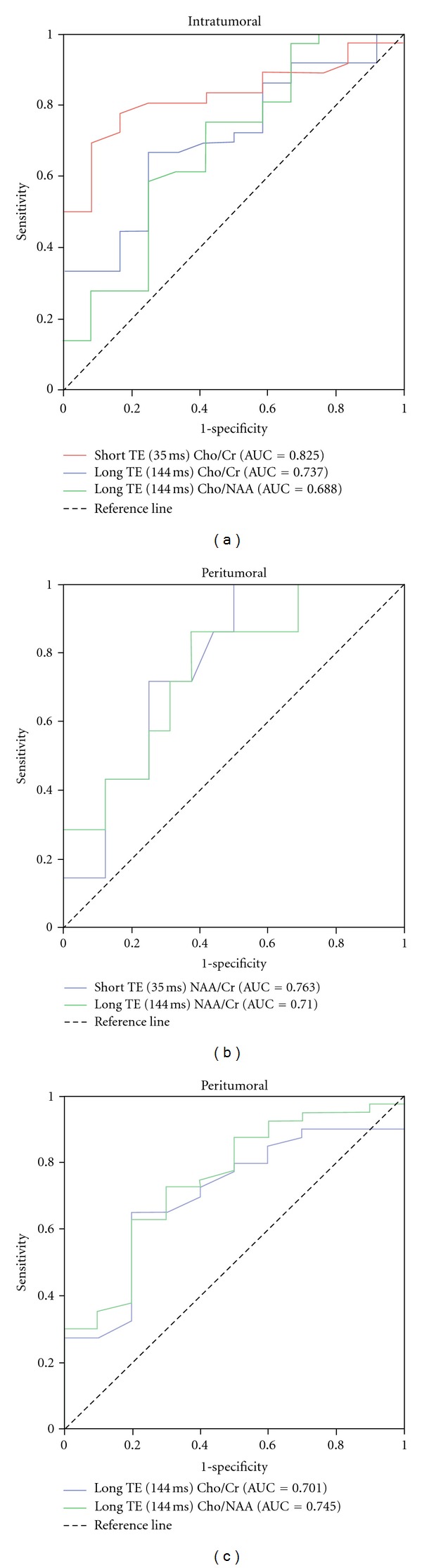
Upper graph (a) illustrates the receiver operating characteristic (ROC) curves of intratumoral short TE Cho/Cr and long TE Cho/Cr and Cho/NAA ratios. Lower graphs illustrate ROC curves of peritumoral short TE NAA/Cr, long TE NAA/Cr (b) and long TE Cho/Cr and Cho/NAA (c) ratios, for differentiating low from high grade gliomas.

ROC analysis was implemented in order to acquire the optimal cut-off values that could potentially differentiate high from low grade gliomas (Figure 6). 

Regarding the intratumoral region of interest, the cut-off values of the metabolic ratios which significantly differentiated the two tumor groups were short TE Cho/Cr = 1.90, long TE Cho/Cr = 2.70 and Cho/NAA = 1.60 (Figure 5). 

Concerning the peritumoral area, the optimal metabolic ratio cut-off values were short TE NAA/Cr = 1.40, long TE NAA/Cr = 1.65, long TE Cho/Cr = 1.20 and Cho/NAA = 1.70.

The resulting sensitivity and specificity of the calculated cut off values are shown in Table 3. The diagnostic sensitivity of peritumoral NAA/Cr at short TE (100%) proved to be superior to that of the other metabolic ratios, whereas the intratumoral Cho/Cr at short TE reached the highest levels of specificity (82%) and diagnostic accuracy (AUC = 0.825).

## 4. Discussion

 It is of paramount importance to differentiate high from lowgrade gliomas before establishing an effective treatment. Conventional MR imaging can be a helpful tool to preoperatively predict glial tumor grade [13, 24, 25]. However, it is possible to observe similar radiological findings for low and high grade gliomas and the distinction among different grades by conventional MR imaging may be difficult and unreliable. 

 Advanced MR imaging techniques, such as ^1^H-MRS, can further improve the diagnostic accuracy during tumor grading [13, 24, 25] up to approximately 15% [26]. ^1^H-MRS provides information on the biochemical profile of a tissue and is increasingly being used as a noninvasive method for the detection and grading of brain tumors [27]. For a reliable ^1^H-MRS procedure, spectroscopic data acquisition should be optimized and thus different parameters should be properly adjusted. 

One of such parameters that can largely influence the spectrum is the echo time. It is well known that when short TE ^1^H-MRS is performed, it is possible to detect metabolites with short T2 relaxation times, and there is little need for T2 corrections. Nevertheless, there are several disadvantages such as the distortion of the spectra baseline under the effects of eddy current, water contamination and the overlapped lipids and lactate peaks, resulting in higher shimming demands. On the contrary, long TE ^1^H-MRS may be chosen to detect the metabolites of longer relaxation times with little or no contamination of residual water, lipids, or fat tissue and thus without baseline distortions. 

Few studies have been devoted to the contribution of both short and long TE ^1^H-MRS for glioma grading, collecting metabolic data solely from the intratumoral areas [14, 22, 23]. However, due to the infiltrative nature of gliomas, the need to spectroscopically investigate their peritumoral areas still remains. 

Towards this direction the current study investigated the contribution of short and long TE ^1^H-MRS at 3T in distinguishing high from low grade gliomas and particularly evaluated the hypothesis that the metabolic findings from the intratumoral as well as the peritumoral areas would provide valuable information regarding glioma grading.

### 4.1. Intratumoral Region

 For both glioma grades, all the metabolic ratios between the intratumoral area and the cNA were significantly different. This observation is in good agreement with previous reports which suggest that the simultaneous elevation of Cho, mI, and LL with NAA reduction is reliable indicator of cerebral gliomas [15, 24].

In the current study, intratumoral short TE Cho/Cr ratio and long TE Cho/Cr and Cho/NAA ratios proved to be strong indicators of tumor grade as they significantly differentiated the two tumor groups. A review of the literature reveals that the significant increase of intratumoral Cho/Cr and LL/Cr ratios in high grade gliomas (III + IV) compared with low grade (II) at either short or long TE are reproducible observations among many previous studies [6, 13, 14, 17, 18, 20–22]. According to the study of Zeng et al. [28] the intratumoral Cho/NAA ratio was significantly different between high and lowgrade groups for long TE (144 msec), which is in agreement with our observations. Additionally, the linear correlation of Cho with Ki-67 labelling index of cellular proliferative activity indeed suggests that Cho may be a string predictor of tumor grade [29]. In agreement with Fountas et al. our study revealed that the NAA/Cr metabolite ratio may not be a reliable malignancy marker either using short or long TE [30]. 

 The presence of necrosis is another important factor for the distinction among glioma grades and hence LL peaks are expected to be prominent in a highly necrotic tumor [31]. The majority of glioblastomas present larger amount of necrosis over gliomas of grade III [14] and thus, the inclusion of necrotic tissue inside a relatively small-sized voxel is sometimes inevitable. Consequently, such inclusion results in higher LL peaks in grade IV tumor spectra compared with those of grade III tumors. This is the reason of the two spectral patterns observed at the present study for the intratumoral area of high grade gliomas. Although quantification and analyses were not performed in this study, lipids may serve as a valuable index for the differentiation of these two tumor groups.

The importance of elevated mI as a putative marker of astrocytomas has been previously noted [32]. In the current study, mI/Cr ratio did not significantly differentiate low from high grade gliomas. Nonetheless, previous studies have reported that mI/Cr ratio tends to increase [14, 32] or decrease with glioma grade [33]. It is well known that elevation of mI may occur either by rapid cellular proliferation or cellular distraction and gliosis from irradiation exposure. The majority of the patients recruited for the aforementioned studies had been previously treated, extracting potentially inaccurate results on predicting glioma grade via mI/Cr ratio. In compliance with our study, Spampinato et al. did not reveal significant differentiation of intratumoral mI/Cr at short TE among untreated patients with low and high grade gliomas [34]. 

 Several authors have demonstrated the usefulness of employing short TE (35 msec) rather than long TE (144 msec) sequences on high-field MR spectroscopy in grading cerebral gliomas, when only the intratumoral region was taken into account [26]. This was also confirmed from our study. On short TE, all high grade gliomas exhibited high LL peaks (Figure 2(b)), while those peaks were within normal ranges in low grade spectra (Figure 2(a)). 

On the contrary, 10 out of 30 high grade gliomas, which revealed high LL peaks on short TE spectra, failed to reveal those peaks when long TE was performed. Additionally, short TE intratumoral Cho/Cr cut-off value revealed the highest levels of sensitivity and specificity for the differentiation of the two tumor groups.

### 4.2. Peritumoral Region

 Regions of altered signal outside the enhancing margins of gliomas represent a variable combination of vasogenic oedema and infiltrating tumor cells due to their ability to invade adjacent cerebral parenchyma [35, 36].

On this basis, we found significantly lower NAA/Cr of both TEs and higher long TE Cho/Cr and Cho/NAA ratios in the peritumoral area of high grade gliomas when compared to low grade gliomas. Hence, the hypothesis that gliomas of higher grade should be more infiltrative than low grade was confirmed. However, 4/52 (8%) high grade gliomas did not reveal evidence of tumor infiltration. Di Costanzo et al. reported that if edema is prominent, the increase of interstitial water “dilutes” the signal of metabolites and produces normal spectra with reduced metabolite peaks [4].

In agreement with our results, Scarabino et al. analyzed the spatial distribution of the metabolites outside gliomas and they successfully discriminated lowgrade from highgrade gliomas. In particular, in highgrade gliomas, Cho was elevated in the peritumoral edema, while NAA was reduced in the periphery [26]. Similarly, Server et al., using long TE (135 msec) ^1^H-MRS, observed elevation in peritumoral Cho/Cr and Cho/NAA metabolic ratios in relation to grading. The peritumoral Cho/NAA and Cho/Cr ratios were significantly higher in high grade than in low grade gliomas [9]. On the contrary, Weber et al. demonstrated pathologic spectra in the peritumoral region of all the gliomas studied with increased peritumoral long TE (135 msec) Cho/Cr and Cho/NAA ratios; however, they did not observe significant alterations of the ratios among glioma grades [8]. 

Overall, the peritumoral area of gliomas proved to be more valuable in predicting glioma grade as more metabolic ratios (4 ratios) significantly differentiated the two tumor groups, over the intratumoral area (3 ratios). Therefore, not only the intratumoral area should be taken into account for glioma grading, but also the peritumoral area.

The potential useful cut-off values of each metabolic ratio were determined to differentiate high from lowgrade gliomas (Table 3). Those results were quite close to previous reports [24, 25, 28]. However, metabolic ratios of gliomas and their threshold values vary among different studies [9, 14, 17, 18, 24, 25, 28, 37]. These variations are caused by the differences in the acquisition parameters, voxel size and location, subject's number, tumor's own heterogeneity, and extrinsic factors such as MR field strength [14]. 

In our study, the sensitivities of intratumoral short and long TE Cho/Cr ratio in glioma grading were 78% and 68%, respectively, indicating moderate true-positive and false-negative rates. However, the corresponding specificities, 82% and 75%, respectively, indicate that the most low grade gliomas will be correctly classified. The sensitivity of long TE intratumoral Cho/NAA ratio was 75%, and hence this metabolic ratio can be useful in determining glioma grade. However, the low specificity (58%) might be due to the high levels of Cho and the low levels of NAA observed in some low grade gliomas. Other researchers such as Law et al. also confirm this result [24]. 

The sensitivities of peritumoral short and long TE NAA/Cr ratios in the differentiation of low grade versus high grade gliomas were 100% and 82%, respectively, demonstrating that this metabolic ratio may be a valuable index in predicting tumor grade. Nevertheless, the low specificities of 50% and 61%, respectively, mean that the false-positive rates are relatively high and the true-negative rates are correspondingly low. In other words, some low-grade gliomas will be falsely identified as high-grade. The sensitivities of peritumoral long TE Cho/Cr and Cho/NAA ratios in the differentiation of the two tumor groups were 64% and 62%, respectively, indicating low true-positive and high false-negative rates. However, the high specificity (80%) means that only few low grade gliomas will be falsely identified as high-grade. 

As Law et al. reported, a high sensitivity is more important in identifying high grade gliomas than a high specificity because of the more serious consequences of false negative findings [24]. Hence, if a single TE must be chosen for the discrimination of low and high grade gliomas, short TE is preferable because it reached higher averaged sensitivity (89%) in predicting glioma grade, over long TE (70%).

One limitation of the current study is that other metabolites such as lactate and Glutamate-Glutamine complex were not evaluated, although it may be possible to provide discrimination of glioma grade [14, 31]. 

As explained above, at single voxel scan techniques, preferably small voxel sizes are chosen, shimming is localized, and water and lipid suppression are better when compared to multivoxel techniques. Thereby, due to higher shimming and water suppression demands on short TE, spectra were acquired using SV techniques. On the contrary, in 2D-MRSI, shimming is global and is more difficult to obtain sufficient water and lipid suppression; however, multiple voxels can be collected during one scan [14, 38]. Therefore, due to the lower shimming demands of long TE and the attenuation of water and lipids on long TEs, field homogeneity was easier to be reached within the larger volume of interest of the multivoxel acquisition technique. Thus, a second potential limitation is that we have evaluated the influence of two TEs using different ^1^H-MRS acquisition techniques. Nevertheless, we chose similar regions among the two techniques for measuring the metabolic ratios.

## 5. Conclusion

Advanced MR imaging techniques are required in many clinical cases where conventional MR imaging fails to differentiate among glioma grades. ^1^H-MR spectroscopy has been incorporated in the clinical routine to provide an insight into the underlying biological characteristics of brain tumors and hence improve the diagnostic accuracy. 

 In the current study the results suggested that the peritumoral area of gliomas may be more valuable in predicting glioma grade as more metabolic ratios significantly differentiated the two tumor groups, over the intratumoral area.

 From a clinical point of view, if a single TE must be chosen for the discrimination of low and high grade gliomas, the short TE is preferable because it reached higher averaged sensitivity in predicting glioma grade, over long TE. However, acquiring spectra at two different TEs would be advisable whenever possible.

## Figures and Tables

**Table 1:** Mean values and standard deviation of intratumoural and peritumoral metabolic ratios for short TE (TE = 35 msec) and long TE (144 msec).

	Intratumoral				Peritumoral			
	NAA/Cr	Cho/Cr	mI/Cr	Cho/NAA	NAA/Cr	Cho/Cr	mI/Cr	Cho/NAA
Short TE (35 msec)								
Low grade gliomas	1.04 ± 0.39	1.52 ± 0.46	0.85 ± 0.24	1.65 ± 1.08	1.65 ± 0.45	0.83 ± 0.15	0.61 ± 0.16	0.51 ± 0.09
High grade gliomas	1.22 ± 0.39	1.90 ± 0.63	0.90 ± 0.35	1.40 ± 0.60	1.30 ± 0.29	1.00 ± 0.27	0.55 ± 0.11	0.79 ± 0.31
*P* values	—	0.02	—	—	0.047	—	—	0.019
Long TE (144 msec)								
Low grade gliomas	1.29 ± 0.53	2.17 ± 0.67	0.34 ± 0.14	2.02 ± 1.20	1.74 ± 0.57	1.26 ± 0.27	0.24 ± 0.09	0.79 ± 0.32
High grade gliomas	1.19 ± 0.44	2.99 ± 1.18	0.40 ± 0.27	2.93 ± 1.72	1.34 ± 0.39	1.58 ± 0.52	0.21 ± 0.14	1.22 ± 0.63
*P* values	—	0.013	—	0.049	0.05	0.05	—	0.016

Note. *P* values denote the metabolite ratio differences among high and low grade gliomas. (—) denotes no statistical difference.

**Table 2:** Mean values and standard deviation of cNA metabolic ratios for short TE (TE = 35 msec) and long TE (144 msec).

	cNA			
	NAA/Cr	Cho/Cr	mI/Cr	Cho/NAA
Short TE (35 msec)				
Low grade gliomas	1.60 ± 0.18	0.81 ± 0.14	0.57 ± 0.05	0.52 ± 0.12
High grade gliomas	1.55 ± 0.24	0.96 ± 0.27	0.62 ± 0.13	0.64 ± 0.34
Long TE (144 msec)				
Low grade gliomas	1.91 ± 0.49	1.16 ± 0.30	0.17 ± 0.05	0.63 ± 0.20
High grade gliomas	1.73 ± 0.48	1.34 ± 0.37	0.18 ± 0.08	0.87 ± 0.50

**Table 3:** Measures of the optimal cut-off values with their corresponding sensitivities and specificities of the metabolic ratios which provide a statistical differentiation between high and low grade gliomas.

Metabolites ratios	Cut-off values	Sensitivity (%)	Specificity (%)	AUC
Intratumoral area				
Short TE Cho/Cr	1.90	78	82	0.825
Long TE Cho/Cr	2.70	68	75	0.737
Long TE Cho/NAA	1.60	75	58	0.688
Peritumoral area				
Short TE NAA/Cr	1.40	100	50	0.763
Long TE NAA/Cr	1.65	82	61	0.710
Long TE Cho/Cr	1.20	64	80	0.701
Long TE Cho/NAA	1.70	62	80	0.745
